# Comparison of *Aspergillus*-specific antibody cut-offs for the diagnosis of aspergillosis

**DOI:** 10.3389/fmicb.2022.1060727

**Published:** 2022-12-06

**Authors:** Chien-Wen Hsiao, Tsai-Hung Yen, Yen-Ching Wu, Jun-Peng Chen, Yun-Yu Chen, Wen-Nan Huang, Yi-Hsing Chen, Yi-Ming Chen

**Affiliations:** ^1^Division of Allergy, Immunology and Rheumatology, Department of Internal Medicine, Taichung Veterans General Hospital, Taichung, Taiwan; ^2^Master Program for Health Administration (EMHA), Department of Industrial Engineering and Enterprise, Tunghai University, Taichung, Taiwan; ^3^Department of Medical Research, Taichung Veterans General Hospital, Taichung, Taiwan; ^4^Cardiovascular Research Center, School of Medicine, National Yang Ming Chiao Tung University, Taipei City, Taiwan; ^5^Heart Rhythm Center, Division of Cardiology, Department of Medicine, Taipei Veterans General Hospital, Taipei City, Taiwan; ^6^Institute of Epidemiology and Preventive Medicine College of Public Health, National Taiwan University, Taipei City, Taiwan; ^7^Cardiovascular Center, Taichung Veterans General Hospital, Taichung, Taiwan; ^8^School of Medicine, National Yang Ming Chiao Tung University, Taipei City, Taiwan; ^9^College of Business and Management, Ling Tung University, Taichung, Taiwan; ^10^Department of Post-Baccalaureate Medicine, College of Medicine, National Chung Hsing University, Taichung, Taiwan; ^11^Institute of Biomedical Science and Rong Hsing Research Center for Translational Medicine, National Chung Hsing University, Taichung, Taiwan

**Keywords:** allergic bronchopulmonary aspergillosis, *Aspergillus*, *Aspergillus* antibody, chronic pulmonary aspergillosis, *Aspergillus fumigatus*, *Aspergillus niger*, ImmunoCAP, invasive aspergillosis

## Abstract

**Background:**

*Aspergillus* diseases are frequently encountered in patients who are immunocompromised. Without a prompt diagnosis, the clinical consequences may be lethal. *Aspergillus*-specific antibodies have been widely used to facilitate the diagnosis of *Aspergillus* diseases. To date, universally standardized cut-off values have not been established. This study aimed to investigate the cut-off values of *Aspergillus*-specific antibodies and perform a narrative review to depict the geographic differences in the Taiwanese population.

**Methods:**

We analyzed enrolled 118 healthy controls, 29 patients with invasive aspergillosis (IA), chronic pulmonary aspergillosis (CPA), and allergic bronchopulmonary aspergillosis (ABPA) and 99 with disease control, who were tested for *Aspergillus fumigatus* and *Aspergillus niger*-specific IgG and IgE using ImmunoCAP. 99 participants not fulfilling the diagnosis of IA, CPA, and ABPA were enrolled in the disease control group. The duration of retrieval of medical records from June 2018 to September 2021. Optimal cut-offs and association were determined using receiver operating characteristic curve (ROC) analysis.

**Results:**

We found that patients with CPA had the highest *A. fumigatus*-specific IgG levels while patients with ABPA had the highest *A. fumigatus*-specific IgE, and *A. niger*-specific IgG and IgE levels. In patients with CPA and ABPA, the optimal cut-offs of *A. fumigatus*-specific IgG and *A. niger*-specific IgG levels were 41.6, 40.8, 38.1, and 69.9 mgA/l, respectively. Geographic differences in the cut-off values of *A. fumigatus*-specific IgG were also noted. Specifically, the levels were different in eco-climatic zones.

**Conclusion:**

We identified the optimal cut-offs of *Aspergillus*-specific antibodies to facilitate a precise diagnosis of aspergillosis. The observed geographic differences of the antibody levels suggest that an eco-climatic-specific reference is needed to facilitate a prompt and accurate diagnosis of aspergillosis.

## Introduction

*Aspergillus* spp. exists in a wide range of environments. A substantial number of species are pathogens that are responsible for a collective group of clinical diseases referred to as aspergillosis (Al-Rahman et al., 2018). Among them, *Aspergillus fumigatus* is the main cause of disease, accounting for approximately 80% of aspergillosis (Al-Rahman et al., 2018; Jat et al., 2018). Other species of *Aspergillus*, such as *Aspergillus niger, Aspergillus flavus*, and *Aspergillus terreus* are less frequently reported in the literature.

Aspergillosis is classified into three types, allergic bronchopulmonary aspergillosis (ABPA), chronic pulmonary aspergillosis (CPA), and invasive aspergillosis (IA), according to host immunity (De Pauw et al., 2008; Agarwal et al., 2013a; Denning et al., 2016; Al-Rahman et al., 2018; Jat et al., 2018; Sehgal et al., 2018). Most patients with aspergillosis present with respiratory symptoms, such as cough, wheezing, chest pain, dyspnea, fever, weight loss, and hemoptysis. However, some patients remain asymptomatic (Agarwal et al., 2013a; Douglass et al., 2014; Jat et al., 2018). If left untreated, irreversible lung damage and fibrosis may occur.

Allergic bronchopulmonary aspergillosis is an allergic inflammatory disease resulting from exposures to *Aspergillus* spp. that is commonly seen in patients with asthma and cystic fibrosis (Watkins et al., 2012; Agarwal et al., 2013a,b, 2015, 2017; Douglass et al., 2014; Shah and Panjabi, 2016; Al-Rahman et al., 2018; Jat et al., 2018). ABPA has been estimated to affect 4 million people globally (Agarwal et al., 2013a,b, 2014, 2017; Douglass et al., 2014; Shah and Panjabi, 2016; Jat et al., 2018). It can take decades from symptom onset to a definite diagnosis of ABPA (Agarwal et al., 2013a; Douglass et al., 2014; Jat et al., 2018). Previous studies showed that in patients with asthma and cystic fibrosis, 1–6% and 2–15% may develop ABPA, respectively (Barton et al., 2008; Watkins et al., 2012; Agarwal et al., 2013a,b, 2015, 2017; Douglass et al., 2014; Shah and Panjabi, 2016). Therefore, ABPA should be considered in patients with refractory and uncontrolled asthma.

Previous studies have indicated that CPA affects 3 million people around the world (Page et al., 2016, 2018; Salzer et al., 2017; Sehgal et al., 2018; Lee et al., 2020). Pulmonary cavities due to prior lung diseases or infection, especially tuberculosis (TB) may increase the likelihood of CPA as the cavities provide a great opportunity for *Aspergillus* infection (Denning et al., 2016; Page et al., 2016; Salzer et al., 2017; Al-Rahman et al., 2018; Sehgal et al., 2018; Jabeen et al., 2020). The mortality rates of CPA can be as high as 50–85% (Fujiuchi et al., 2016; Lee et al., 2020). Even with prompt treatment, 20–50% of patients with CPA may still mortality (Salzer et al., 2017). Importantly, ABPA/CPA is often indistinguishable from TB (Agarwal et al., 2013a; Jat et al., 2018). Therefore, a definitive diagnostic tool is needed to confirm the diagnosis of ABPA/CPA.

In patients who are immunocompromised, such as those with acquired immune deficiency syndrome, hematologic malignancy, and patient’s post-organ transplant who require immunosuppressants. IA is a common opportunistic infection (Al-Abdely et al., 2014). Compared with ABPA and CPA, the mortality rate of IA may be up to 50% (Cadena et al., 2016). A prompt diagnosis and appropriate timely therapy are crucial to patients with IA (De Pauw et al., 2008; Fujiuchi et al., 2016; Lee et al., 2020).

In the diagnosis of aspergillosis, evidence of *Aspergillus* infection needs to be confirmed by clinical presentations, radiographic manifestations, and laboratory findings. There is not a single test that can definitively confirm the diagnosis of aspergillosis (Agarwal et al., 2013a,b, 2015, 2017; Baxter et al., 2013; Denning et al., 2016; Page et al., 2016; Shah and Panjabi, 2016; Salzer et al., 2017; Al-Rahman et al., 2018; Lee et al., 2021). *Aspergillus*-specific IgG is one of the most essential tests for the diagnosis of CPA (Denning et al., 2016; Sehgal et al., 2018; Lee et al., 2021). Although the biological reference for *Aspergillus*-specific IgG has been extensively investigated to determine the best biological reference range for aspergillosis, a universal consensus has not been reached. Previous studies have highlighted that the optimal cut-offs of *Aspergillus*-specific antibody tests could vary due to ethnicity, geographic location, climate differences, and exposure frequency (Van Hoeyveld et al., 2006; Agarwal et al., 2013a, 2014; Sehgal et al., 2018; Jabeen et al., 2020; Lee et al., 2020, 2021). As such, a universal unified cut-off does not exist. The suitable reference range of *Aspergillus*-specific antibody tests should be determined locally (Lee et al., 2020, 2021).

A previous study from Taiwan found that *A. niger* was the most frequently isolated *Aspergillus* spp. (26.5%) (Hsiue et al., 2012). In addition, *A. fumigatus* was the leading causative pathogen of invasive aspergillosis (14.7%) in Taiwan (Hsiue et al., 2012). Therefore, our study aimed to investigate the optimal cut-off values of *A. fumigatus*- and *A. niger-*specific antibodies for the diagnosis of ABPA, CPA, and IA in Taiwan and compare these with previous reports to investigate the geographic variations.

## Materials and methods

### Study participants

This study included 118 healthy controls and 128 participants (29 with aspergillosis, 99 with disease control) who visited the outpatient clinic or were admitted to the inpatient ward of Taichung Veterans General Hospital and underwent examination for *Aspergillus-*specific IgG and IgE antibodies from June 2018 to September 2021. Of these patients, 6 met the diagnostic criteria for IA of the European Organization for Research and Treatment of Cancer/Invasive Fungal Infections Cooperative Group and the National Institute of Allergy and Infectious Disease Mycoses Study Group consensus group (De Pauw et al., 2008; Al-Abdely et al., 2014) according to the following criteria: (a) host factors (history of neutropenia, corticosteroids or recognized T cell immunosuppressants, inherited severe immunodeficiency); (b) clinical features (lower respiratory tract fungal disease, tracheobronchitis, sinonasal, or CNS infection); and (c) mycological evidence; 18 were diagnosed with CPA using the diagnostic criteria for CPA as per the European Society for Clinical Microbiology and Infectious Disease and European Respiratory Society guidelines (Denning et al., 2016) fulfilling all the following criteria for at least 3 months: (a) one or more pulmonary cavities on the thoracic imaging; (b) direct evidence of *Aspergillus* infection or immunological response to *Aspergillus*; and (c) exclusion of alternative diagnoses; 5 were diagnosed with ABPA according to the diagnostic criteria for ABPA from the International Society of Human and Animal Mycology working group (Agarwal et al., 2013a) with the following criteria: (a) asthma or cystic fibrosis; (b) *A. fumigatus*-specific IgE > 0.35 KUA/L; (c) total IgE > 1,000 KU/L; and two of the following criteria: (a) present of precipitating or IgG antibodies against *A. fumigatus* (b) radiographic pulmonary opacities consistent with ABPA (c) total eosinophil count > 500 cells/μL. Participants tested for *Aspergillus-*specific IgG and IgE antibodies tests but not fulfilling the diagnosis of IA, CPA, and ABPA were enrolled in the disease control group. Healthy participants with no self-reported TB or asthma were enrolled in the healthy control group. This study was approved by the Ethics Committee of Clinical Research, Taichung Veterans General Hospital (IRB no. CE21478A). As patient data were anonymized before analysis, the need for written consent was waived.

### Measurement of *Aspergillus*-specific IgG, IgE, and galactomannan antigen test

*Aspergillus fumigatus-* and *A. niger-*specific IgG and IgE detection with Fluorescence Enzyme Immunoassay was performed by using the ImmunoCAP system (Phadia, Uppsala, Sweden), *Aspergillus* specific IgE ≥ 0.35 KUA/L was considered positive. Galactomannan antigen detection with ELISA was performed by using the Bio-Rad Platelia *Aspergillus* Antigen (Bio-Rad, Marnes-la-Coquette, France). Galactomannan antigen test ≥ 0.5 index was considered positive.

### Data collection

Clinical data and comorbidities were extracted from the electric health record of Taichung Veterans General Hospital. The participants’ age and laboratory test results for alanine aminotransferase and creatinine levels were documented at the time of clinical diagnosis of IA, CPA, ABPA for the aspergillosis group, and when *Aspergillus*-specific IgG and IgE antibodies tests were performed for the disease control and healthy control groups. Comorbidities, including asthma (ICD-9: 493.x; ICD-10: J45.x), chronic obstructive pulmonary disease (COPD) (ICD-9: 496; ICD-10: J44.x), autoimmune disease (ICD-9:710.x, 714; ICD-10: M30-M36), chronic kidney disease (ICD-9: 585.x; ICD-10:N18.X), diabetes mellitus (250.x; ICD-10: E08-E13), tuberculosis (TB) (ICD-9: 010-018; ICD-10: A15-A19), and malignancy (ICD-9: 140.x – 208.x; ICD-10: C00-D49) were determined using ICD-9/ICD-10 codes, and performed at least twice in the outpatient system or once in the inpatient system.

### Narrative review of *Aspergillus*-specific IgG in previous studies

A narrative literature review of previous studies regarding *Aspergillus*-specific IgG was performed using the following keyword: “*Aspergillus*,” “Cut-off,” “ImmunoCAP,” and “Human” to search in Pubmed.

### Statistical analysis

Data were expressed as medians (inter-quartile ranges) or numbers (percentages). The selected parameters were compared among the *Aspergillus* diseases group, disease control group, and healthy control group, and analyzed using the Chi-square test or Kruskal–Wallis test. Area under the curve was measured using receiver operating characteristics curve (ROC) analyses in the disease control group and patients with aspergillosis. Optimal cut-offs of *A. fumigatus*- and *A. niger*-specific IgG and IgE for the diagnosis was determined by DeLong method. All statistical analyses were performed using the Statistical Package for the Social Science, version 22.0 (SPSS, IBM Corp., Armonk, NY, USA) and MedCalc^®^ Statistical Software version 20.014 (MedCalc Software Ltd., Ostend, Belgium). *P*-value < 0.05 was considered statistically significance.

## Results

### Comparing the demographic data and comorbidities of the enrolled participants

Demographic data of the diseases (IA, CPA, and ABPA), disease control, and healthy control groups are shown in Table 1. Patients with aspergillosis were significantly older compared with the healthy controls. Patients with ABPA exhibited the highest *A. fumigatus*- and *A. niger-*specific IgE, compared with their counterparts. Moreover, *A. fumigatus*- and *A. niger-*specific IgG were higher in the ABPA and CPA groups, compared with those with IA, and the healthy and disease controls. In contrast, galactomannan antigen levels were similar in all groups. Asthma was observed in all patients with ABPA, and the prevalence of asthma among those with diseases was higher than the healthy controls. Moreover, COPD and TB were more frequently observed in CPA patients relative to healthy controls. Furthermore, there was a higher proportion of cancer patients in the IA group compared with the healthy control group.

**TABLE 1:** Comparing the demographic data of patients with aspergillosis, the disease controls, and the healthy controls.

	IA (*n* = 6)		CPA (*n* = 18)		ABPA (*n* = 5)		Disease controls (*n* = 99)		Healthy controls (*n* = 118)		*P*-value
Age	65.5	(42.7–73.2)	65.5	(58.0–72.0)	58.0	(39.0–63.5)	66.0	(58.0-73.0)	42.0	(35.0–53.0)	< 0.001^d^*
Sex											0.069
Female	2	(33.3%)	5	(27.8%)	3	(60.0%)	57	(57.6%)	71	(61.7%)	
Male	4	(66.7%)	13	(72.2%)	2	(40.0%)	42	(42.4%)	44	(38.3%)	
*A. f*-specific IgE (KUA/l)	0.01	(0.01–0.09)	0.11	(0.04–1.27)	0.82	(0.62–21.75)	0.01	(0.01–0.03)	0.01	(0.01–0.01)	< 0.001^acdef^
*A. f-*specific IgG (mgA/l)	29.15	(8.22–109.70)	89.70	(59.68–144.00)	77.10	(48.50–155.50)	23.20	(12.60–40.20)	29.80	(18.05–55.55)	< 0.001^cde^
*A. n*-specific IgE (KUA/l)	0.01	(0.01–0.02)	0.03	(0.01–0.82)	0.44	(0.26–11.80)	0.01	(0.01–0.01)	0.01	(0.01–0.01)	< 0.001^acdef^
*A. n*-specific IgG (mgA/l)	19.35	(6.09–45.93)	56.90	(43.23–82.05)	71.80	(22.65–190.00)	18.10	(8.88–31.00)	14.90	(8.06–32.93)	< 0.001^cd^
Galactomannan antigen (index)	0.19	(0.12–3.34)	0.13	(0.09–0.23)	0.10	(0.05–0.14)	0.11	(0.07–0.21)	0.13	(0.13–0.13)	0.393
Creatinine (mg/dL)	1.07	(0.49–4.52)	0.86	(0.68–1.15)	0.80	(0.70–1.23)	0.80	(0.70–1.01)	0.80	(0.70–1.00)	0.768
ALT (U/L)	22.0	(10.5–27.5)	17.5	(13.7–23.0)	18.0	(5.0–63.0)	18.0	(15.0–29.5)	16.5	(12.0–24.7)	0.128
Asthma	1	(16.7%)	5	(27.8%)	5	(100%)	35	(35.4%)	4	(3.4%)	< 0.001^dfg^
Autoimmune diseases	0	(0.0%)	2	(11.1%)	1	(20.0%)	23	(23.2%)	8	(6.8%)	0.009^g^
CKD	1	(16.7%)	3	(16.7%)	1	(20.0%)	18	(18.2%)	1	(0.8%)	< 0.001^g^
COPD	2	(33.3%)	11	(61.1%)	0	(0.0%)	35	(35.4%)	0	(0.0%)	< 0.001^bdg^
DM	1	(16.7%)	2	(11.1%)	1	(20.0%)	17	(17.2%)	6	(5.1%)	0.069
Malignancy	4	(66.7%)	6	(33.3%)	0	(0.0%)	31	(31.3%)	7	(5.9%)	< 0.001^bdg^
TB	3	(50.0%)	8	(44.4%)	1	(20.0%)	21	(21.2%)	0	(0.0%)	< 0.001^bdg^

Data expressed as median (interquartile range), *p*-value by Chi-square test or Kruskal–Wallis test. *Post-hoc* analysis *p*<0.05, ^a^IA vs. ABPA; ^b^IA vs. Healthy controls; ^c^CPA vs. Disease controls; ^d^CPA vs. Healthy controls; ^e^ABPA vs. Disease controls; ^f^ABPA vs. Healthy controls; ^g^Disease controls vs. Healthy controls; IA, invasive aspergillosis; CPA, chronic pulmonary aspergillosis; ABPA, allergic bronchopulmonary aspergillosis; *A. f, Aspergillus fumigatus; A. n, Aspergillus niger;* ALT, alanine aminotransferase; CKD, chronic kidney disease; COPD, chronic obstructive pulmonary disease; DM, diabetes mellitus; TB, tuberculosis.

### Optimal levels of *Aspergillus*-specific antibodies for the diagnosis of aspergillosis

**FIGURE 1:**
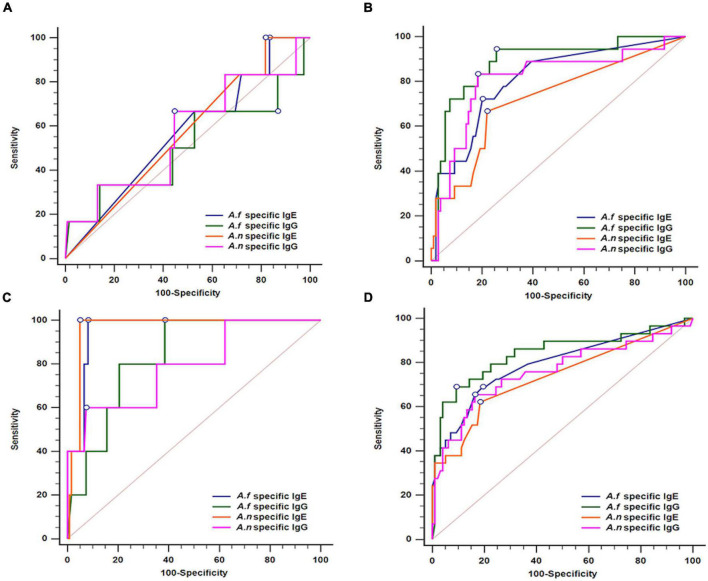
Receiver operating characteristic curve (ROC) analyses of *Aspergillus fumigatus*- and *Aspergillus niger*-specific IgG and IgE for the diagnosis of **(A)** Invasive aspergillosis, **(B)** chronic pulmonary aspergillosis, **(C)** allergic bronchopulmonary aspergillosis, and **(D)** all of the above; *A. f, Aspergillus fumigatus*; *A. n, Aspergillus niger*.

To determine the optimal cut-off values of *Aspergillus*-specific antibodies for the diagnosis of aspergillosis, we performed ROC analysis (Table 2 and Figure 1). In the CPA and ABPA groups, a decent diagnostic ability of *A. fumigatus*- and *A. niger*-specific IgG and IgE were demonstrated (AUC, ranging from 0.723 to 0.966). Moreover, *A. fumigatus*- and *A. niger*-specific IgG exhibited a higher AUC than IgE tests in distinguishing patients with CPA (Figure 1B). Likewise, the IgE tests outperform IgG tests in patients with ABPA (Figure 1C) with marked increased positive likelihood ratios. However, the optimal cut-offs of *A. fumigatus*- and *A. niger*-specific IgG and IgE could not be determined in the IA group (Table 2 and Figure 1A), indicating that diagnostic tests other than *Aspergillus* antibodies may be necessary for the diagnosis of IA. As seen in Figure 1D, *A. fumigatus*-specific IgG appeared to be a better test for identifying a composite outcome of IA, CPA, and ABPA than counterparts. However, specifically in IA, CPA, and ABPA groups, the AUCs between anti-*A. fumigatus* and anti-*A. niger* antibodies were similar.

**TABLE 2:** Receiver operating characteristic curve (ROC) analyses of *Aspergillus fumigatus*- and *Aspergillus niger*-specific IgG and IgE for the diagnosis of aspergillosis.

Variables	AUC	(95% CI)	*p*	*p* for comparision*	Optimal cut-offs	Youden index	Sensitivity	Specificity	LR +	LR-
**A. Invasive aspergillosis**										
*A. f*-specific IgE	0.567	(0.476–0.654)	0.546	0.994	≤0.18	0.17	100.00%	16.53%	1.20	0.00
*A. n*-specific IgE	0.567	(0.477–0.655)	0.400		≤0.06	0.18	100.00%	18.18%	1.22	0.00
*A. f*-specific IgG	0.508	(0.418–0.598)	0.959	0.850	>9.73	0.20	66.67%	13.11%	0.77	2.54
*A. n*-specific IgG	0.564	(0.473–0.651)	0.654		≤20	0.22	66.67%	54.92%	1.48	0.61
**B. Chronic pulmonary aspergillosis**										
*A. f*-specific IgE	0.808	(0.729–0.873)	<0.001	0.126	>0.06	0.52	72.22%	79.82%	3.58	0.35
*A. n*-specific IgE	0.723	(0.637–0.799)	<0.001		>0.01	0.45	66.67%	77.98%	3.03	0.43
*A. f*-specific IgG	0.887	(0.819–0.936)	<0.001	0.069	>41.6	0.69	94.44%	74.55%	3.71	0.08
*A. n*-specific IgG	0.811	(0.732–0.874)	<0.001		>40.8	0.65	83.33%	81.82%	4.58	0.20
**C. Allergic bronchopulmonary aspergillosis**										
*A. f*-specific IgE	0.957	(0.906–0.985)	<0.001	0.612	>0.28	0.92	100.00%	91.80%	12.20	0.00
*A. n*-specific IgE	0.966	(0.917–0.990)	<0.001		>0.18	0.95	100.00%	95.08%	20.33	0.00
*A. f*-specific IgG	0.836	(0.760–0.895)	<0.001	0.509	>38.1	0.62	100.00%	61.79%	2.62	0.00
*A. n*-specific IgG	0.793	(0.712–0.859)	0.017*		>69.9	0.53	60.00%	92.68%	8.20	0.43
**D. Invasive aspergillosis/Chronic pulmonary aspergillosis/Allergic bronchopulmonary aspergillosis**										
*A. f*-specific IgE	0.794	(0.713–0.860)	<0.001	0.165	>0.05	0.50	68.97%	80.61%	3.56	0.38
*A. n*-specific IgE	0.737	(0.651–0.811)	<0.001		>0.01	0.44	62.07%	81.63%	3.38	0.46
*A. f*-specific IgG	0.841	(0.766–0.899)	<0.001	0.013*	>58.1	0.60	68.97%	90.91%	7.59	0.34
*A. n*-specific IgG	0.761	(0.677–0.832)	<0.001		>40.8	0.49	65.52%	83.84%	4.05	0.41

*P-value was compared using DeLong’s method; ROC, receiver operating characteristic curve; *A. f, Aspergillus fumigatus; A. n: Aspergillus niger;* AUC, area under curve; LR +, positive likelihood ratio; LR-, negative likelihood ratio.

### A narrative review of *Aspergillus*-specific IgG for the diagnosis of aspergillosis

**FIGURE 2:**
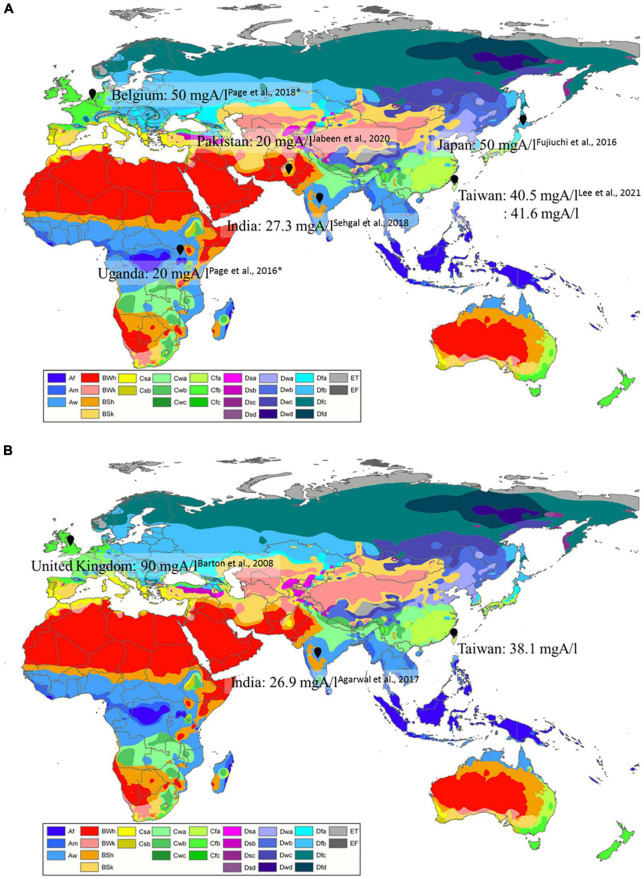
Geographic differences of **(A)** *Aspergillus fumigatus*-specific IgG cut-off levels in patients with CPA, **(B)** *A. fumigatus*-specific IgG cut-off levels in patients with ABPA by eco-climatic zones. CPA, chronic pulmonary aspergillosis; ABPA, allergic bronchopulmonary aspergillosis. *Cases from the United Kingdom. Created with updated world map of the Köppen–Geiger climate classification (Peel et al., 2007). Description of Köppen climate symbols (Supplementary Table 1).

To investigate the association of *Aspergillus-*specific IgG cut-offs, ethnicity, and geographic differences, we summarized data from previous studies and our results (Table 3). We also depicted the eco-climatic-specific cut-offs using the world map of Köppen–Geiger climate classification (Peel et al., 2007; Figure 2). Interestingly, *A. fumigatus*-specific IgG cut-offs in patients with CPA seemed to be different in climate zones. Figure 2A. For example, the lowest cut-offs were from Uganda, India, and Pakistan (20–27.3 mgA/l), which belong to tropical or arid climate, followed by Taiwan (40.5–41.6 mgA/l, sub-tropical climate); the highest in Japan and Belgium (both 50 mgA/l, temperate, and cold climate). A similar trend was observed in the *A. fumigatus*-specific IgG levels from patients with ABPA (Figure 2B). The lowest cut-offs were found in an Indian study (26.9 mgA/l), followed by our results (38.1 mgA/l), and British data (90 mgA/l). Based on these differences, we suggest that eco-climatic-specific *A. fumigatus*-specific IgG cut-offs may be required as references.

**TABLE 3:** A narrative review of *Aspergillus*-specific IgG in previous studies.

	*Aspergillus* species	Study population	*N*	Cut-off (mgA/l)	Sensitivity/Specificity (%)	Country	References
1	*A. fumigatus*	Healthy controls	121	2–68.7		Omani	Al-Rahman et al., 2018
2	*A. fumigatus*	Healthy controls	120	2.79–66.45		South Africa	Watkins et al., 2012
3	*A. fumigatus*	AFAA	48	26.9	88/100	India	Agarwal et al., 2017
		ABPA	102				
4	*A. fumigatus*	Control	59	90	91/88	United Kingdom	Barton et al., 2008
		ABPA	7				
		*A. fumigatus* sensitized	21				
5	*A. fumigatus*	Healthy controls	21	20	80.95/82.05	Pakistan	Jabeen et al., 2020
		Disease control	18				
		CPA	21				
	*A. flavus*	Healthy controls	21	30	80.95/79.49		
		Disease control	18				
		CPA	21				
6	*A. fumigatus*	CCPA with fungal ball	103	27.3	95.6/100	India	Sehgal et al., 2018
		CCPA without fungal ball	34				
		Control	50				
7	*A. fumigatus*	Healthy control	122	50	98/84	Japan	Fujiuchi et al., 2016
		Disease control	51				
		CPA	96				
8	*A. fumigatus*	CPA (UK)	241	20	96/98	United Kingdom	Page et al., 2016
		Healthy controls (Uganda)	100				
9	*A. fumigatus*	CPA (United Kingdom)	241	50	83.8/95.6	United Kingdom	Page et al., 2018
		Healthy controls (Belgium)	114				
10	*A. fumigatus*	CPA	21	40.5	86.7/80.2	Taiwan	Lee et al., 2021
		Non-CPA	241				
11	*A. fumigatus*	CPA	116	40	97/none	UK	Baxter et al., 2013
		ABPA/SAFS	46				
		Other	13				
12	*A. fumigatus*	Healthy control	118			Taiwan	
		Disease control	99				
		IA	6	9.73	66.67/13.11		
		CPA	18	41.6	94.44/74.55		
		ABPA	5	38.1	100/61.79		
		IA/CPA/ABPA	29	58.1	68.97/90.91		
	*A. niger*	Healthy control	118			Taiwan	
		Disease control	99				
		IA	6	20	66.67/54.92		
		CPA	18	40.8	83.33/81.82		
		ABPA	5	69.9	60/92.68		
		IA/CPA/ABPA	29	40.8	65.52/83.84		

*A. fumigatus, Aspergillus fumigatus; A. niger, Aspergillus niger; A. flavus, Aspergillus flavus;* ABPA, allergic bronchopulmonary aspergillosis; AFAA, *A. fumigatus*-associated asthma; CPA, chronic pulmonary aspergillosis; CCPA, chronic cavitary pulmonary aspergillosis; SAFS, asthma with fungal sensitization; IA, invasive aspergillosis.

## Discussion

In this study, we established the optimal cut-offs of *A. fumigatus-* and *A. niger-*specific IgG for the diagnosis of aspergillosis. We also observed geographic differences in the cut-off values of *Aspergillus* specific IgG for patients with CPA and ABPA. Our results suggested that a climate type normal range is needed for an accurate and precise diagnosis of aspergillosis.

Traditionally, the diagnosis of aspergillosis relied primarily on clinical manifestations, radiographic findings, and either direct evidence from fungal culture or indirect evidence from serology tests. Although these diagnostic modalities cannot be completely replaced, *Aspergillus*-specific IgG has substantially improved the sensitivity, reproducibility, and subjective interpretation. Moreover, it is less time-consuming compared with fungal culture, precipitin, and galactomannan antigen tests (Van Hoeyveld et al., 2006; Baxter et al., 2013; Page et al., 2016; Jat et al., 2018; Sehgal et al., 2018). *Aspergillus*-specific IgE ≥ 0.35 KUA/L and *Aspergillus*-specific IgG cut-offs ≥ 40 mgA/l were considered positive accordingly to manufacturer’s recommendations (Barton et al., 2008; Agarwal et al., 2013a; Page et al., 2016). In contrast, our results revealed that *Aspergillus*-specific IgG may vary in patients with different *Aspergillus* diseases. By using a Taiwanese hospital-based population with aspergillosis, this study provided a practical reference of *Aspergillus*-specific IgG for countries around this region with same climate type.

Previous studies have demonstrated that ethnicity groups, geographic region, and fungal exposure frequency, may contribute to the variations in *Aspergillus*-specific IgG cut-offs (Van Hoeyveld et al., 2006; Agarwal et al., 2013a, 2014; Sehgal et al., 2018; Jabeen et al., 2020). Our study is the first to clearly illustrate that *A. fumigatus*-specific IgG cut-offs for CPA and ABPA varied in different eco-climatic zones. Our result (41.6 mgA/l) concurred with the findings of another Taiwanese study (40.5 mgA/l) that analyzed data from three hospitals across the island (Lee et al., 2021). We originally speculated that temperature may play a crucial role. In tropical or arid countries, such as Uganda, Pakistan, and India, the lower cut-offs may reflect a less robust *Aspergillus*-specific immune reaction in the population compared with those living in the temperate or cold countries, such as Japan and Belgium. On the contrary, a previous study reported that *Aspergillus* increased from the north to the south (Ding et al., 2015). We hypothesized that immune tolerance, with less abundant B cell immunity upon stimulation with a higher dose of the allergen, may play a role in the variation of *Aspergillus*-specific immune reactions. Differences in the comparative population among studies could also affect the calculated cut-off values. Moreover, hygiene hypothesis, or cross-reaction of *Aspergillus*-specific antibodies could be potential confounding factors (Cummings et al., 2007; Okada et al., 2010). Further studies are needed to verify our findings.

In patients who are immunocompromised, such as those with acute leukemia, organ transplantation, and stem cell transplantation, IA may account for 9–32% of the opportunistic infections, and the mortality rate can be as high as 30–60% (Al-Abdely et al., 2014). In this study, malignancy coexisted in two-thirds of patients with IA (66.7%). In contrast to CPA and ABPA, our result failed to demonstrate a diagnostic value for *Aspergillus*-specific antibody tests in the IA population. Microbiology evidence, galactomannan test, β-D-glucan, and microscopic exams are essential for the diagnosis of IA (Agarwal et al., 2015). Interestingly, all patients in our IA group had negative galactomannan tests. This may be due to the selection bias during the process of retrieval and analysis of medical records. Taken together, our results did not support the use of *A. fumigatus* and *A. niger*-specific IgG and IgE for the diagnosis of IA.

Chronic respiratory diseases, such as TB and COPD, may contribute to the development of CPA. The structural airway defects in these conditions may facilitate *Aspergillus* sp. invasion (Salzer et al., 2017; Al-Rahman et al., 2018; Sehgal et al., 2018). CPA can only be diagnosed after respiratory symptoms have been present for more than 3 months, and other medical conditions have been excluded. Our results demonstrated that the AUC of *Aspergillus*-specific IgG was higher than the *Aspergillus*-specific IgE, which was in concordance with the current diagnostic criteria of CPA (Denning et al., 2016). Moreover, many previous studies confirmed *A. fumigatus* as the main causal pathogen (Page et al., 2016, 2018; Al-Rahman et al., 2018; Sehgal et al., 2018; Lee et al., 2020). A previous study found that *A. niger* was the most frequently isolated *Aspergillus* spp. (26.5%) in Taiwan (Hsiue et al., 2012). In this study, we also determined the optimal cut-offs of *A. niger*-specific antibodies, which were similar to the *A. fumigatus*-specific antibodies. Further studies are needed to verify its clinical application.

In our study, we demonstrated that *A. niger-*specific IgE could be a diagnostic tool for ABPA. This has never been reported before. Previous studies found that high correlation between *A. fumigatus*- and *A. niger*-specific IgG in Taiwan (Lee et al., 2020). Consistent with our results, previous studies have also suggested that the *A. fumigatus-*specific IgE level was a sensitive and fundamental test for ABPA (Agarwal et al., 2013b, 2014, 2017). We also found an association between *Aspergillus*-specific IgG and ABPA. A prior study showed that *Aspergillus*-specific IgG could be observed in 69–90% of patients with ABPA. Of note, extremely high levels of *Aspergillus*-specific IgG, and the presence of pulmonary fibrosis or cavitation, strongly suggest that CPA may be progressing (Agarwal et al., 2013a). Future studies are needed to delineate the application of *Aspergillus*-specific IgG in patients with ABPA.

Our study has some limitations. First, the case number of IA and ABPA were small, which prevented a robust analysis of the specific *Aspergillus* disease group. The cutoffs could be different if the enrolled population is increased. Second, the disease control group was consisted with a mixture of patients with comorbidities who were tested for *Aspergillus*-specific antibodies. Moreover, some relevant data, such as smoking habits, was missing. Third, data from the American continent are lacking. We postulated that the cut-offs for *Aspergillus*-specific antibodies across the American continent might be similar to cut-offs from other continent with similar eco-climatic zones. Future study is needed to confirm our assumptions.

In conclusion, we established Taiwan-specific, optimal cut-offs of *Aspergillus*-specific antibodies for the diagnosis of aspergillosis. Geographic variations affected the antibody levels. This suggest that every country should determine its own reference range to ensure a sensitive and precise diagnostic test for aspergillosis.

## Data availability statement

The raw data supporting the conclusions of this article will be made available by the authors, without undue reservation.

## Ethics statement

The studies involving human participants were reviewed and approved by Ethics Committee of Clinical Research, Taichung Veterans General Hospital. Written informed consent for participation was not required for this study in accordance with the national legislation and the institutional requirements.

## Author contributions

C-WH involved in conceptualization of this study, methodology, original draft preparation, review, and editing of the manuscript. T-HY involved in the methodology, data analysis, review, and editing of the manuscript. Y-CW involved in the study design, methodology review, and editing of the manuscript. J-PC involved in the data curation, statistical analysis, review, and editing of the manuscript. Y-YC involved in the data curation, statistical analysis, review, and editing of the manuscript. W-NH involved in the interpretation of the results, resources acquisition, review, and editing of the manuscript. Y-HC participated in the study design, methodology, data interpretation, resources acquisition, review, and editing of the manuscript. Y-MC involved in conceptualization of this study, methodology, data generation, curation, resources acquisition, original draft preparation, review, and editing of the manuscript. All authors have read and agreed to the published version of the manuscript.

## References

[B1] Agarwal R. Aggarwal A. N. Garg M. Saikia B. Chakrabarti A. (2014). Cut-off values of serum IgE (total and *A. fumigatus* -specific) and eosinophil count in differentiating allergic bronchopulmonary aspergillosis from asthma. *Mycoses* 57 659–663. 10.1111/myc.12214 24963741

[B2] Agarwal R. Aggarwal A. N. Sehgal I. S. Dhooria S. Behera D. Chakrabarti A. (2015). Performance of serum galactomannan in patients with allergic bronchopulmonary aspergillosis. *Mycoses* 58 408–412. 10.1111/myc.12334 25959212

[B3] Agarwal R. Chakrabarti A. Shah A. Gupta D. Meis J. F. Guleria R. (2013a). Allergic bronchopulmonary aspergillosis: Review of literature and proposal of new diagnostic and classification criteria. *Clin. Exp. Allergy* 43 850–873. 10.1111/cea.12141 23889240

[B4] Agarwal R. Dua D. Choudhary H. Aggarwal A. N. Sehgal I. S. Dhooria S. (2017). Role of *Aspergillus fumigatus*-specific IgG in diagnosis and monitoring treatment response in allergic bronchopulmonary aspergillosis. *Mycoses* 60 33–39. 10.1111/myc.12541 27523578

[B5] Agarwal R. Maskey D. Aggarwal A. N. Saikia B. Garg M. Gupta D. (2013b). Diagnostic performance of various tests and criteria employed in allergic bronchopulmonary aspergillosis: A latent class analysis. *PLoS One* 8:e61105. 10.1371/journal.pone.0061105 23593402 PMC3625190

[B6] Al-Abdely H. M. Alothman A. F. Salman J. A. Al-Musawi T. Almaslamani M. Butt A. A. (2014). Clinical practice guidelines for the treatment of invasive *Aspergillus* infections in adults in the Middle East region: Expert panel recommendations. *J. Infect. Public Health* 7 20–31. 10.1016/j.jiph.2013.08.003 24029495

[B7] Al-Rahman M. Al Kindi M. Kutty I. Al-Kalbani I. Alshekaili J. (2018). Determination of an *Aspergillus fumigatus*-specific immunoglobulin G reference range in an adult omani population. *Sultan Qaboos Univ. Med. J.* 18 e43–e46. 10.18295/squmj.2018.18.01.007 29666680 PMC5892812

[B8] Barton R. C. Hobson R. P. Denton M. Peckham D. Brownlee K. Conway S. (2008). Serologic diagnosis of allergic bronchopulmonary aspergillosis in patients with cystic fibrosis through the detection of immunoglobulin G to *Aspergillus fumigatus*. *Diagn. Microbiol. Infect. Dis.* 62 287–291. 10.1016/j.diagmicrobio.2008.06.018 18947811

[B9] Baxter C. G. Denning D. W. Jones A. M. Todd A. Moore C. B. Richardson M. D. (2013). Performance of two *Aspergillus* IgG EIA assays compared with the precipitin test in chronic and allergic aspergillosis. *Clin. Microbiol. Infect.* 19 E197–E204. 10.1111/1469-0691.12133 23331929

[B10] Cadena J. Thompson G. R. III Patterson T. F. (2016). Invasive aspergillosis: Current strategies for diagnosis and management. *Infect. Dis. Clin. North Am.* 30 125–142. 10.1016/j.idc.2015.10.015 26897064

[B11] Cummings J. R. Jamison G. R. Boudreaux J. W. Howles M. J. Walsh T. J. Hayden R. T. (2007). Cross-reactivity of non-*Aspergillus* fungal species in the *Aspergillus galactomannan* enzyme immunoassay. *Diagn. Microbiol. Infect. Dis.* 59 113–115. 10.1016/j.diagmicrobio.2007.04.022 17662550

[B12] De Pauw B. Walsh T. J. Donnelly J. P. Stevens D. A. Edwards J. E. Calandra T. (2008). Revised definitions of invasive fungal disease from the European Organization for Research and Treatment of Cancer/Invasive Fungal Infections Cooperative Group and the National Institute of Allergy and Infectious Diseases Mycoses Study Group (EORTC/MSG) Consensus Group. *Clin. Infect. Dis.* 46 1813–1821. 10.1086/588660 18462102 PMC2671227

[B13] Denning D. W. Cadranel J. Beigelman-Aubry C. Ader F. Chakrabarti A. Blot S. (2016). Chronic pulmonary aspergillosis: Rationale and clinical guidelines for diagnosis and management. *Eur. Respir. J.* 47 45–68. 10.1183/13993003.00583-2015 26699723

[B14] Ding N. Xing F. Liu X. Selvaraj J. N. Wang L. Zhao Y. (2015). Variation in fungal microbiome (mycobiome) and aflatoxin in stored in-shell peanuts at four different areas of China. *Front. Microbiol.* 6:1055. 10.3389/fmicb.2015.01055 26557107 PMC4614231

[B15] Douglass J. A. Sandrini A. Holgate S. T. O’Hehir R. E. (2014). “Allergic bronchopulmonary aspergillosis and hypersensitivity pneumonitis,” in *Middleton’s allergy principles and practice*, 8th Edn, eds Adkinson N. F. Bochner B. S. Burks A. W. Busse W. W. Holgate S. T. Lemanske R. F. (Philadelphia, PA: Elsevier/Saunders), 1000–1013.

[B16] Fujiuchi S. Fujita Y. Suzuki H. Doushita K. Kuroda H. Takahashi M. (2016). Evaluation of a quantitative serological assay for diagnosing chronic pulmonary aspergillosis. *J. Clin. Microbiol.* 54 1496–1499. 10.1128/jcm.01475-15 27008878 PMC4879291

[B17] Hsiue H. C. Wu T. H. Chang T. C. Hsiue Y. C. Huang Y. T. Lee P. I. (2012). Culture-positive invasive aspergillosis in a medical center in Taiwan, 2000-2009. *Eur. J. Clin. Microbiol. Infect. Dis.* 31 1319–1326. 10.1007/s10096-011-1445-1 21997774

[B18] Jabeen K. Farooqi J. Iqbal N. Wahab K. Irfan M. (2020). *Aspergillus fumigatus* and *Aspergillus flavus*-specific IgG cut-offs for the diagnosis of chronic pulmonary aspergillosis in Pakistan. *J. Fungi* 6:246. 10.3390/jof6040249 33114653 PMC7711809

[B19] Jat K. R. Vaidya P. C. Mathew J. L. Jondhale S. Singh M. (2018). Childhood allergic bronchopulmonary aspergillosis. *Lung India* 35 499–507. 10.4103/lungindia.lungindia_216_18 30381560 PMC6219146

[B20] Lee M. R. Huang H. L. Chen L. C. Yang H. C. Ko J. C. Cheng M. H. (2020). Seroprevalence of *Aspergillus* IgG and disease prevalence of chronic pulmonary aspergillosis in a country with intermediate burden of tuberculosis: A prospective observational study. *Clin. Microbiol. Infect.* 26 1091.e1–1091.e7. 10.1016/j.cmi.2019.12.009 31901491

[B21] Lee M. R. Huang H. L. Keng L. T. Chang H. L. Sheu C. C. Fu P. K. (2021). Establishing *Aspergillus*-specific IgG cut-off level for chronic pulmonary aspergillosis diagnosis: Multicenter prospective cohort study. *J. Fungi* 7:249. 10.3390/jof7060480 34204844 PMC8231598

[B22] Okada H. Kuhn C. Feillet H. Bach J. F. (2010). The ‘hygiene hypothesis’ for autoimmune and allergic diseases: An update. *Clin. Exp. Immunol.* 160 1–9. 10.1111/j.1365-2249.2010.04139.x 20415844 PMC2841828

[B23] Page I. D. Baxter C. Hennequin C. Richardson M. D. van Hoeyveld E. van Toorenenbergen A. W. (2018). Receiver operating characteristic curve analysis of four *Aspergillus*-specific IgG assays for the diagnosis of chronic pulmonary aspergillosis. *Diagn Microbiol. Infect. Dis.* 91 47–51. 10.1016/j.diagmicrobio.2018.01.001 29398462

[B24] Page I. D. Richardson M. D. Denning D. W. (2016). Comparison of six *Aspergillus*-specific IgG assays for the diagnosis of chronic pulmonary aspergillosis (CPA). *J. Infect.* 72 240–249. 10.1016/j.jinf.2015.11.003 26680697

[B25] Peel M. C. Finlayson B. L. McMahon T. A. (2007). Updated world map of the Köppen-Geiger climate classification. *Hydrol. Earth Syst. Sci.* 11 1633–1644. 10.5194/hess-11-1633-2007

[B26] Salzer H. J. Heyckendorf J. Kalsdorf B. Rolling T. Lange C. (2017). Characterization of patients with chronic pulmonary aspergillosis according to the new ESCMID/ERS/ECMM and IDSA guidelines. *Mycoses* 60 136–142. 10.1111/myc.12589 27910139

[B27] Sehgal I. S. Choudhary H. Dhooria S. Aggarwal A. N. Garg M. Chakrabarti A. (2018). Diagnostic cut-off of *Aspergillus fumigatus*-specific IgG in the diagnosis of chronic pulmonary aspergillosis. *Mycoses* 61 770–776. 10.1111/myc.12815 29920796

[B28] Shah A. Panjabi C. (2016). Allergic bronchopulmonary aspergillosis: A perplexing clinical entity. *Allergy Asthma Immunol. Res.* 8 282–297. 10.4168/aair.2016.8.4.282 27126721 PMC4853505

[B29] Van Hoeyveld E. Dupont L. Bossuyt X. (2006). Quantification of IgG antibodies to *Aspergillus fumigatus* and pigeon antigens by ImmunoCAP technology: An alternative to the precipitation technique? *Clin. Chem.* 52 1785–1793. 10.1373/clinchem.2006.067546 16858079

[B30] Watkins M. L. Kotze E. Benjamin R. L. Hawarden D. (2012). Reference range for specific IgG antibodies to *Aspergillus fumigatus* in the South African adult population. *Curr. Allergy Clin. Immunol.* 25 212–214.

