# Supplementary material for: Comparison of Aspergillus-specific antibody cut-offs for the diagnosis of aspergillosis
Source: Front Microbiol. 2022 Dec 6;13:1060727. doi: 10.3389/fmicb.2022.1060727 (PMC9763268; doi:10.3389/fmicb.2022.1060727)
Supplement: Supplementary file 1 [file Table_1.DOCX]

**Supplementary Table 1.** Description of Köppen climate symbols

| 1st | 2nd | 3rd | Description |
| --- | --- | --- | --- |
| A |  |  | Tropical |
|  | f |  | - Rainforest |
|  | m |  | - Monsoon |
|  | w |  | - Savannah |
| B |  |  | Arid |
|  | W |  | - Desert |
|  | S |  | - Steppe |
|  |  | h | - Hot |
|  |  | k | - Cold |
| C |  |  | Temperate |
|  | s |  | - Dry Summer |
|  | w |  | - Dry Winter |
|  | f |  | - Without dry season |
|  |  | a | - Hot Summer |
|  |  | b | - Warm Summer |
|  |  | c | - Cold Summer |
| D |  |  | Cold |
|  | s |  | - Dry Summer |
|  | w |  | - Dry Winter |
|  | f |  | - Without dry season |
|  |  | a | - Hot Summer |
|  |  | b | - Warm Summer |
|  |  | c | - Cold Summer |
|  |  | d | - Very Cold Winter |
| E |  |  | Polar |
|  | T |  | - Tundra |
|  | F |  | - Frost |
